# Organic amendments and antibiotic resistance gene diversity in tropical agroecosystems of Réunion Island: an exploratory study

**DOI:** 10.1016/j.onehlt.2026.101645

**Published:** 2026-11-04

**Authors:** Rieux Adrien, Dolivet-Maréchal Marion, Doizy Anna, Chiroleu Frederic, Mottes Charles, Soti Valérie, Darnaudery Marie, N. Bravin Matthieu, Cardinale Eric, Baldet Thierry, Doelsch Emmanuel

**Affiliations:** a CIRAD, UMR PVBMT, F-97410 Saint-Pierre, La Réunion, France; b Université Claude Bernard Lyon 1, Laboratoire d'Ecologie Microbienne, UMR CNRS 5557, UMR INRAE 1418, VetAgro Sup, 69622 Villeurbanne, France; c DoAna Statistiques Réunion, F-97480 Saint-Joseph, La Réunion, France; d CIRAD, UPR HortSys, F-97410 Saint-Pierre, La Réunion, France; e CIRAD, UPR AIDA, F-97410 Saint-Pierre, La Réunion, France.; f CIRAD, UR Recyclage et risque, Montpellier F-34398, France; g Recyclage et Risque, Université de Montpellier, Montpellier, France; h CIRAD, UMR ASTRE, Plateforme Technologique CYROI, Sainte-Clotilde, France; i ANSES, National Health Agency, Paris, France

**Keywords:** Antimicrobial resistance, Antibiotic resistance genes, Organic amendments, Environmental resistome, One Health, Tropical agroecosystems

## Abstract

Environmental reservoirs of antimicrobial resistance (AMR) are increasingly recognized as important components of One Health surveillance. We explored antibiotic resistance genes (ARGs) across agricultural environments on Réunion Island using high-throughput qPCR targeting 349 ARGs in 16 samples comprising manure, soils, water and vegetables. ARGs were widespread, with the highest richness and relative abundance in manure-amended soils and manure. ARG abundance was on average approximately 5-fold higher in amended than paired control soils. Among 60 ARGs classified as high risk to human health, 52 were detected, including 16 in water and 22 in raw vegetables. ARG profiles differed primarily among matrices. Antibiotic residues were detected in only five samples, and neither their occurrence nor Cu/Zn concentrations consistently mirrored ARG patterns. This exploratory screening identifies organically amended soils as compartments of particular interest and provides a baseline for targeted One Health surveillance of environmental AMR in tropical agroecosystems.

## Highlights

• High-throughput qPCR revealed diverse ARGs across tropical agricultural matrices.
• Organic amendment was on average associated with ∼5-fold higher soil ARG abundance.
• Fifty-two of 60 high-risk ARGs were detected across sampled environments.
• High-risk ARGs occurred in water and raw vegetables.
• Findings support targeted One Health AMR surveillance in tropical agroecosystems.

## 1 Introduction

Antimicrobial resistance (AMR) is a major global health threat whose dynamics extend beyond clinical and veterinary settings to environmental reservoirs [1], [2]. Agricultural systems are particularly relevant because antibiotic-resistant bacteria and antibiotic resistance genes (ARGs) can enter soils through manure, wastewater and other organic amendments, potentially contributing to their persistence and redistribution across environmental compartments [3], [4], [5], [6]. In addition, copper and zinc, two feed additives that accumulate in animal manure, may further contribute to resistance selection and persistence through co-selection mechanisms [7].

These processes are particularly relevant from a One Health perspective because agricultural soils, livestock, water and food constitute interconnected interfaces between environmental, animal and human microbiomes [2], [8]. However, environmental AMR remains poorly characterized in tropical agroecosystems.

Réunion Island is a French tropical territory in the southwestern Indian Ocean combining livestock and crop production within a densely populated insular environment. AMR has previously been documented in human, livestock and aquatic compartments on the island [9], [10], [11], but ARG occurrence within agricultural environments remains largely unexplored.

We therefore conducted an exploratory screening of ARGs across contrasting agricultural matrices on Réunion Island, with particular emphasis on differences between organically amended and control soils and on the occurrence of ARGs of potential One Health relevance. Antibiotic residue and trace element concentrations were additionally investigated as potential environmental covariates.

## 2 Materials and methods

Sixteen samples were collected in June 2022 from three agricultural settings and four natural areas across Réunion Island (Fig. 1; Table S1). Samples comprised manure or compost (*n* = 4), agricultural soils (*n* = 6), vegetables (*n* = 1) and water (*n* = 1), together with four soils from natural areas. Agricultural soils included organically amended and unamended plots. Manure originated from pig and poultry production, including fresh and composted material. Soil and manure samples consisted of pooled subsamples, while water was obtained through composite stream-water sampling. Each composite environmental sample was considered an individual observational unit. Subsamples collected within a given sampling location were pooled before analysis and were not considered independent biological replicates. The sampling design was exploratory and included unequal numbers of samples across environmental matrices. Detailed procedures are provided in Supplementary Methods S1.

**Fig. 1:**
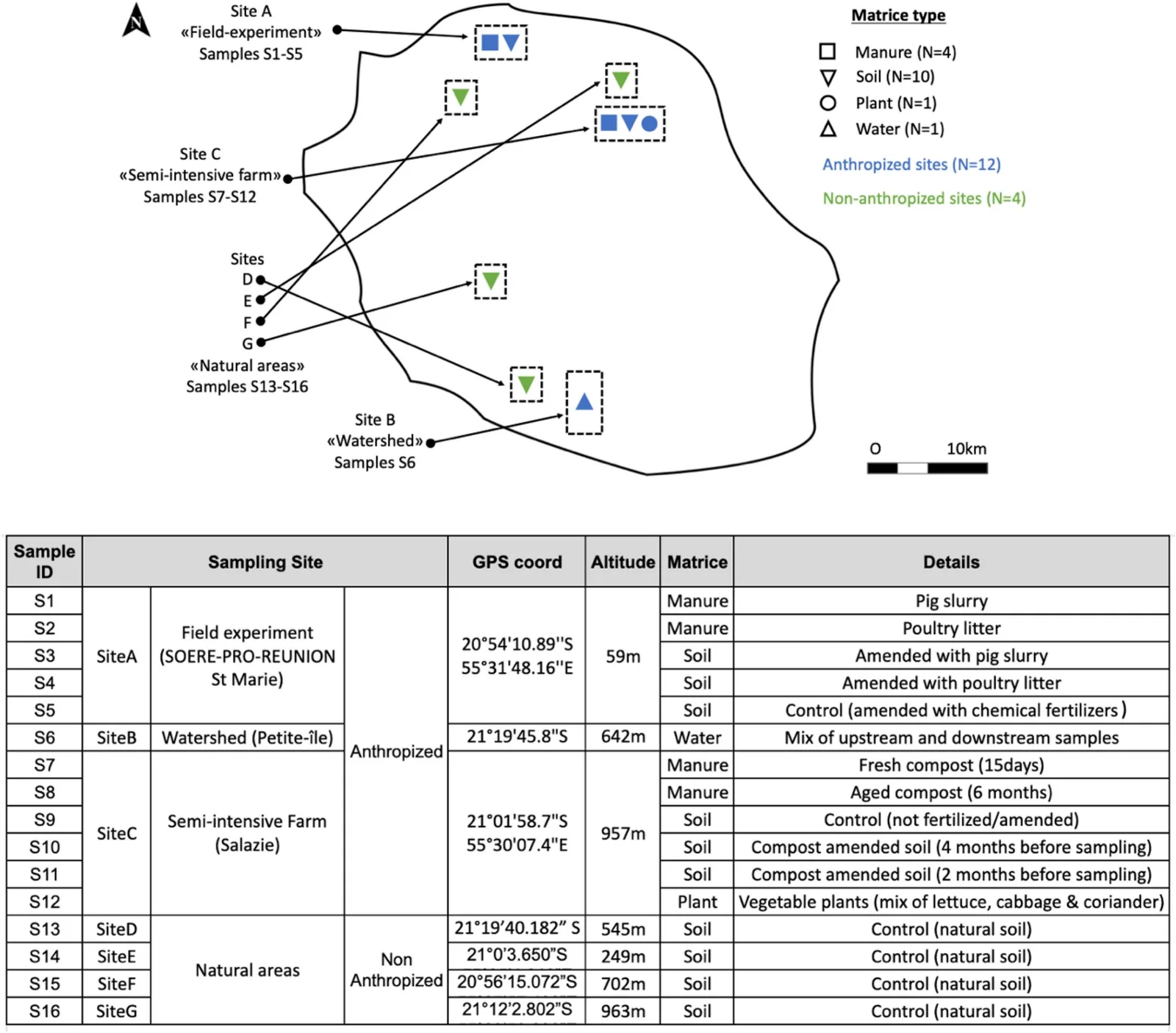
Description of sampling sites and environmental samples collected across Réunion Island. Sampling included manure/compost, agricultural and natural soils, water and vegetable material collected across three agricultural settings and four natural areas. Detailed sample characteristics are provided in Table S1.

DNA was extracted using matrix-specific commercial kits and standardized prior to SmartChip qPCR analysis, as detailed in the Supplementary Methods. High-throughput qPCR targeted 349 ARGs representing ten antibiotic classes, together with 22 mobile genetic elements (MGEs, including integrons) and taxonomic/control markers (Supplementary Methods S2; Table S5). Sixty ARGs were classified as high risk to human health according to Zhang et al. [12]. Following the qPCR quality-control procedures detailed in the Supplementary Methods, detection was based on a Ct threshold of 27 and relative abundance was calculated using the comparative Ct method normalized to 16S rRNA [13]. Mean ARG richness and relative abundance, together with their 95% confidence intervals, were estimated for each matrix using nonparametric bootstrap resampling of sample-level values rather than individual ARG assays. Samples were additionally screened for 52 antibiotic residues and six trace elements (Supplementary Methods S3–S4).

ARG profiles were explored using Bray–Curtis dissimilarities and non-metric multidimensional scaling (NMDS). Fold changes in ARG relative abundance were calculated for four amended/control soil comparisons (S3/S5, S4/S5, S10/S9, and S11/S9). For each ARG, fold changes were averaged across comparisons, and the overall mean enrichment was calculated as the arithmetic mean of these ARG-specific values. Additional exploratory comparisons of composting and time following amendment were also performed (Supplementary Methods S2 & S5).

## 3 Results and discussion

A total of 296 ARGs and 14 MGEs were detected in at least one sample, with an average of 186 ARGs and 11 MGEs detected per sample on average (Table S5, Fig. S2). ARG richness differed among matrices, with manure and amended soils containing the highest numbers of detected ARGs and water the lowest (Fig. 2A). Relative abundance showed a similar pattern: amended soils exhibited the highest mean abundance relative to 16S rRNA (0.021), followed by manure (0.010), control soils (0.004), vegetables (0.003) and water (0.0008) (Fig. 2B, Fig. 3B, Fig. S4). Matrix-specific ARG detections are summarized in Table S2. Ancillary analyses of taxonomic markers and comparisons between anthropized and natural control soils are provided in Figs. S1 and S3, respectively.

**Fig. 2:**
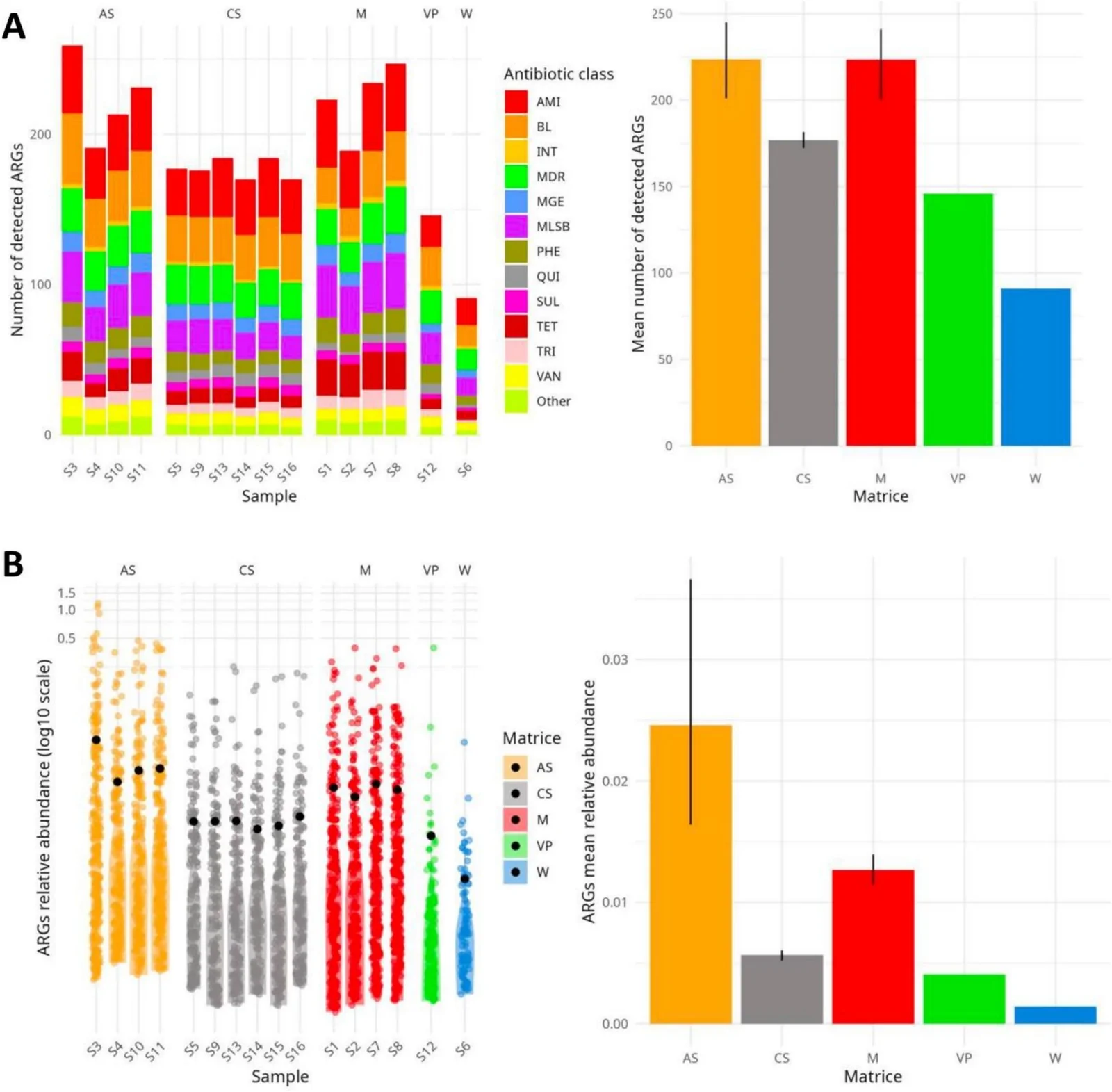
ARG richness and relative abundance across environmental matrices. (A) Number of detected ARGs in individual samples (left) and mean ARG richness per matrix (right). (B) Relative abundance of individual detected ARGs normalized to the 16S rRNA gene (left), with black dots indicating the arithmetic mean for each sample. Bars represent the mean ARG relative abundance per matrix, calculated from sample-level means (right). Error bars indicate 95% confidence intervals estimated by nonparametric bootstrap resampling of composite samples. Confidence intervals are not shown for matrices represented by a single sample (*n* = 1). AS, amended soils; CS, control soils; M, manure; VP, vegetable plants; W, water.

**Fig. 3:**
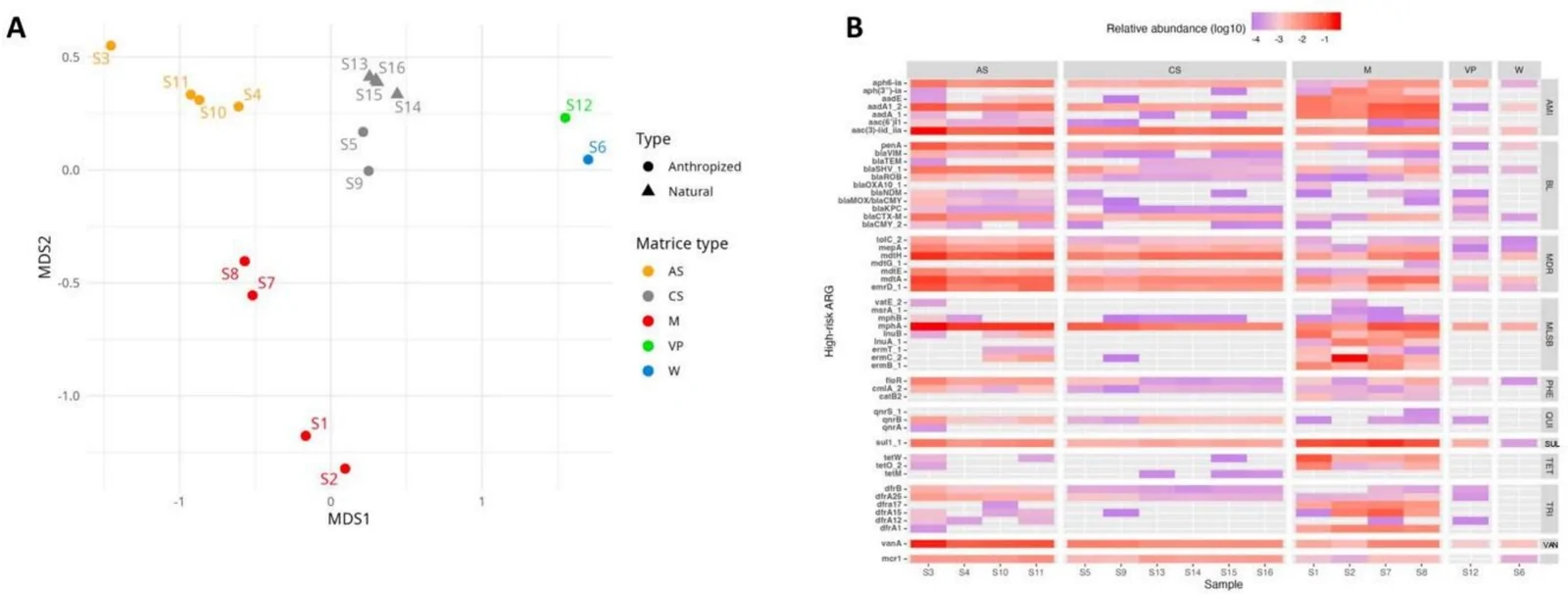
ARG profiles across contrasting agricultural matrices. (A) NMDS ordination based on Bray–Curtis dissimilarities of ARG relative-abundance profiles. (B) Heatmap of the relative abundance of the 60 ARGs classified as high risk to human health according to Zhang et al. [12]. The complete heatmap of detected resistance targets is provided in Fig. S4. AS, amended soils; CS, control soils; M, manure; VP, vegetable plants; W, water.

Interestingly, a substantial diversity of ARGs was also detected in soils from natural areas with limited apparent anthropogenic influence (Fig. 2A, Fig. S3). This observation raises questions about the natural background diversity of antibiotic resistance determinants in tropical soils, which remains poorly characterized. Although the origin of these ARGs cannot be established from our data, this finding highlights the importance of considering natural soil resistomes when assessing the contribution of agricultural practices to environmental antibiotic resistance.

The association with organic amendment was particularly apparent in within-site comparisons. ARG relative abundance was approximately 5-fold higher on average in amended than paired unamended soils, with increases reaching approximately 75-fold for individual ARGs (Fig. 2B, Fig. S5C). This pattern is consistent with previous studies showing enrichment of soil resistomes following manure application [3], [5], [14], [15]. Additional comparisons suggested decreases in mean ARG abundance during composting (Fig. S5A) and between two and four months following amendment (Fig. S5B).

From a One Health perspective, 52 of the 60 ARGs (86%) classified as high risk to human health were detected in at least one sample, and 14 occurred across all sampled matrix types (Fig. 3B; Fig. S4, Table S5). Sixteen high-risk ARGs were detected in the water sample and 22 in pooled raw vegetable material (Fig. 3B; Table S5). Because water and vegetables were each represented by a single composite sample, these observations cannot establish prevalence or transmission pathways. Nevertheless, water and fresh produce constitute relevant environmental interfaces for potential human exposure [16], [17], making these detections useful for prioritizing future surveillance. ARGs detected jointly in manure and amended soils while absent from paired control soils are listed in Table S3. These co-occurrence patterns should not be interpreted as evidence of transmission. Importantly, qPCR detection does not identify bacterial hosts and does not demonstrate ARG mobility, resistance expression, presence of viable resistant pathogens or transmission to humans.

ARG composition also varied among environmental matrices. NMDS ordination separated manure, soils, vegetables and water, while amended soils tended to separate from control soils (Fig. 3A). Given the limited and unbalanced design, these patterns cannot formally disentangle matrix from site effects. They are nevertheless consistent with evidence that physicochemical conditions and microbial community composition are major determinants of environmental resistomes [18], [19].

Antibiotic residues were detected in only five of the 16 samples: pig slurry, poultry litter, aged compost and two agricultural soils (Table S4). No residues were detected in water, vegetables or natural soils (Table S4). The limited number of positive samples precluded robust statistical assessment of associations between antibiotic concentrations and ARG abundance (Fig. S7). Similarly, Cu and Zn concentrations were not significantly associated with ARG richness or relative abundance (Figs. S6 and S8). Thus, current detectable concentrations of antibiotics or Cu/Zn did not explain the observed ARG patterns. Persistence following historical inputs, microbial community composition and horizontal gene transfer represent non-exclusive processes that could contribute to ARG maintenance [7], [20].

This study should be considered an exploratory environmental screening rather than a representative survey of Réunion Island agroecosystems. The limited number of samples, single sampling period and unbalanced representation of matrices restrict inference beyond the investigated sites. In particular, some matrices were represented by a single composite sample, and comparisons between amended and unamended soils involved shared controls, limiting the independence of these comparisons and preventing robust statistical inference**.** High-throughput qPCR additionally detects targeted resistance determinants without identifying their bacterial hosts, genomic context, mobility or phenotypic expression. Detection of ARGs classified as high-risk does not, by itself, establish a direct human-health risk. Importantly, ARG relative abundances were normalized to the 16S rRNA gene and do not represent absolute ARG loads. Differences in normalized abundance may reflect changes in ARG copy numbers, bacterial community composition, or variation in 16S rRNA gene copy numbers among taxa. These factors cannot be disentangled from the present data. Altogether, these limitations prevent inference of transmission pathways or direct human-health risks. Nevertheless, the broad ARG diversity observed and their approximately 5-fold higher abundance in organically amended than paired control soils identify agricultural environments as relevant targets for future and more extensive One Health AMR investigations.

## 4 Conclusions and One Health implications

This first exploratory screening revealed a broad diversity of ARGs across agricultural and environmental matrices on Réunion Island, with the strongest signal in manure and organically amended soils. ARG abundance was approximately 5-fold higher in amended than paired control soils, while high-risk ARGs were detected across several matrices. These findings identify agricultural, environmental and food-associated compartments warranting targeted investigation. Larger longitudinal studies combining representative sampling with metagenomics and culture-based approaches will be required to identify bacterial hosts, characterize mobile resistance determinants and assess potential transmission pathways. These results provide a baseline for designing such One Health surveillance in tropical agroecosystems.

## CRediT authorship contribution statement

**Rieux Adrien:** Visualization, Validation, Supervision, Software, Resources, Project administration, Methodology, Investigation, Funding acquisition, Formal analysis, Data curation, Conceptualization, Writing – review & editing, Writing – original draft. **Dolivet-Maréchal Marion:** Visualization, Resources, Methodology, Formal analysis, Data curation, Writing – original draft. **Doizy Anna:** Visualization, Software, Methodology, Investigation, Conceptualization, Writing – review & editing. **Chiroleu Frederic:** Validation, Supervision, Software, Writing – review & editing. **Mottes Charles:** Supervision, Methodology, Writing – review & editing. **Soti Valérie:** Supervision, Methodology, Writing – review & editing. **Darnaudery Marie:** Supervision, Methodology. **N. Bravin Matthieu:** Supervision, Project administration, Methodology, Investigation, Funding acquisition, Conceptualization, Writing – review & editing. **Cardinale Eric:** Project administration, Funding acquisition, Conceptualization, Writing – review & editing. **Baldet Thierry:** Supervision, Project administration, Funding acquisition, Writing – review & editing. **Doelsch Emmanuel:** Validation, Supervision, Project administration, Methodology, Investigation, Funding acquisition, Formal analysis, Data curation, Conceptualization, Writing – review & editing, Writing – original draft.

## Funding

This work was co-funded by the European Union and the Région Réunion. L'Europe s'engage à La Réunion avec the FEDER (contract 2024-1248-005756). It also received support from the 10.13039/501100014141European Agricultural Fund for Rural Development (EAFRD Health and Biodiversity and SADUR programs), co-financed by the 10.13039/501100000780European Union, the State and the Réunion Region. Europe is committed to Réunion with the EAFRD, for which the Réunion Department is the managing authority. This work was also marginally supported for samples from Site A by Veolia Water within the framework of the project “Boues Grand Prado”.

## Declaration of competing interest

The authors declare the following financial interests/personal relationships which may be considered as potential competing interests: E. Doelsch and M.N. Bravin received financial support from Veolia Water. The other authors declare no competing interests.

## Data Availability

The raw qPCR output data, along with the R code used for data analysis and figure generation, are available at: https://gitlab.cirad.fr/pvbmt/2026_AMR_RUN
