# Supplementary material for: Organic amendments and antibiotic resistance gene diversity in tropical agroecosystems of Réunion Island: an exploratory study
Source: One Health. 2026 Nov 4;23:101645. doi: 10.1016/j.onehlt.2026.101645 (PMC13763005; doi:10.1016/j.onehlt.2026.101645)
Supplement: Supplementary file 1 [file mmc1.pdf]

SUPPLEMENTARY MATERIAL - Organic amendments and antibiotic resistance gene diversity in tropical soils of Réunion Island: An exploratory study

A- Supplementary Methods

S1. Study sites and sampling

Réunion Island is a volcanic island constituting a French overseas territory with an area of 2,512 km² and a population of approximately 900,000 inhabitants, located in the southwestern Indian Ocean, 679 km from Madagascar and 170 km from Mauritius. Réunion Island has a tropical climate with hot, rainy summers from December to April and cooler, drier winters from May to November.

Sixteen samples were collected in June 2022 from different matrices and agroecosystems (Table S1). **Site A** (SOERE PRO Réunion) is a 1-ha long-term field trial established in 2014 and located in Sainte-Marie. The trial investigates the partial substitution of imported synthetic fertilizers by locally produced organic fertilizers in a sugarcane agroecosystem. Samples comprised pig slurry (S1), poultry litter (S2), soil amended with pig slurry (S3), soil amended with poultry litter (S4), and a control soil fertilized only with synthetic fertilizers (S5). **Site B** is an approximately 850-ha watershed located in Petite-Île and comprising around one hundred smallholder farms practicing livestock farming, crop production including grassland, market-gardening crops and sugarcane, or both. A composite stream-water sample (S6) was obtained from this watershed. **Site C**, located in Salazie, is a semi-intensive farm in an area characterized by intensive livestock activity, where poultry and pigs are bred and animal manure is applied to vegetable-production areas. Samples comprised fresh manure aged for approximately 15 days (S7) and six-month composted manure (S8), both originating from the same manure batch; an unamended agricultural soil (S9); soils sampled four months (S10) and two months (S11) after compost application from the same agricultural plot receiving the same amendment; and vegetable material (S12) collected from compost-amended plots. Four

additional **control** soil samples (S13–S16) were collected from natural areas located on the edge of or within Réunion National Park and without recent agricultural use.

For soil sampling, six subsamples were collected from the surface layer (0–10 cm) at different locations within each plot using a hand auger, pooled and sieved through a 4-mm mesh. Manure samples similarly consisted of six subsamples taken from different locations within each manure source and mixed thoroughly. Water samples were collected throughout week 20 of 2022 from two automated stations positioned upstream and downstream of the watershed. Each station collected stream-water subsamples every 1 h 15 min, which were pooled to generate weekly composite samples. A total volume of 3 L from both stations was pooled and filtered through a 0.2-μm mixed-cellulose-ester membrane filter under a laminar flow hood. For vegetable samples, aerial green tissues were collected from fresh lettuce, coriander and cabbage leaves, chopped and homogenized. All materials were handled with sterile gloves, equipment was disinfected with 99% isopropyl alcohol between samples, and samples were transported on ice and stored at −20°C until molecular and chemical analyses.

S2. DNA extraction and high-throughput qPCR

DNA was extracted from manure and soil samples using the Qiagen DNeasy PowerSoil Pro Kit, using 250 mg and 50 mg of material, respectively. Qiagen Plant Pro Kit and PowerWater Kit were used to extract DNA from 100 mg of vegetable material and water filters, respectively. All extractions were performed according to the manufacturers' protocols and DNA was stored at −20°C until analysis. A total of 384 primer sets were used [S1], with the complete list and corresponding primer sequences provided in **Excel File S1**. These included 349 ARGs conferring resistance to ten major antibiotic classes (aminoglycosides (AMI), β-lactams (BL), multidrug resistance (MDR), macrolide-lincosamide-streptogramin B (MLSB), phenicols (PHE), quinolones (QUI), sulfonamides (SUL), tetracyclines (TET), trimethoprim (TRI) and vancomycin (VAN)), 22 mobile genetic elements (MGEs, including integrons), ten genes associated with microbial taxonomic groups and three 16S rRNA control genes. Sixty ARGs were classified as high risk to human health according to Zhang et al. [12]. When multiple variants of a gene classified as high-risk were targeted by the SmartChip assays, all variants were included (see **Excel File S1**).

High-throughput qPCR was performed by Resistomap (https://www.resistomap.com/) using the SmartChip Real-Time PCR system (Takara Bio, CA, USA). DNA extracts were adjusted to a target concentration of 10 ng/µL before SmartChip analysis, corresponding to approximately 0.2 ng of DNA per reaction. Each DNA sample was analysed in three technical replicates. Reactions were performed in 100 nL volumes containing 20 nL of template DNA, SmartChip TB Green Gene Expression Master Mix, 300 nM of each forward and reverse primer, and qPCR-grade water. Cycling conditions consisted of initial denaturation at 95°C for 10 min, followed by 40 cycles of denaturation at 95°C for 15 s, annealing at 60°C for 15 s, and extension at 72°C for 30 s. Melting-curve analysis was performed at the end of amplification, with the temperature increasing to 97°C in increments of 0.4°C. Assay-specific melting-temperature (Tm) acceptance criteria are provided in **Excel File S1**.

Amplification quality was assessed using melting-curve profiles and fluorescence-derived amplification factors estimated near the quantification cycle (Cq, also referred to as Ct). These amplification factors differ from efficiencies determined using dilution-series standard curves. Reactions showing nonspecific melting curves, multiple peaks, or evidence of primer-dimer formation based on melting-temperature information from no-template controls (NTCs) were excluded. Reactions with amplification factors outside the acceptable range of 1.7–2.3 were also discarded. Technical replicate concordance was subsequently assessed. When the Ct standard deviation among three replicates exceeded 0.5 cycles, the most divergent replicate was excluded. When three replicates remained after the initial filtering procedure, the replicate with an amplification factor furthest from the range of 1.9–2.1 was excluded. A mean Ct value was calculated only when at least two valid technical replicates remained, with a Ct standard deviation ≤1 cycle. Assays with fewer than two valid replicates were considered nondetected. Internal positive and negative controls were included in the SmartChip qPCR workflow. Sample data were validated only when the corresponding controls met the internal quality-control acceptance criteria.

A Ct threshold of <27 was applied for positive gene detection, following Stedtfeld et al. (2018) [ref S1]. Amplification signals with Ct values ≥27 were considered negative for detection. Relative gene abundance (R) was calculated relative to the 16S rRNA gene using the comparative Ct method [ref S2]:

$R = 2^{-\Delta Ct}$

with:

$\Delta Ct = Ct(ARG) - Ct(16S\ rRNA).$

For paired samples, fold changes in ARG abundance were calculated using the comparative ΔΔCt method:

$FC = 2^{-\Delta\Delta Ct}.$

To calculate the average fold change in ARG relative abundance between amended and unamended soils, four comparisons were performed (S3/S5, S4/S5, S10/S9, and S11/S9). For each ARG, the mean fold change was calculated across the available comparisons. The overall mean fold change was then obtained by averaging these ARG-specific mean fold changes.

S3. Antibiotic residue analysis

Samples were screened for 52 antibiotic residues encompassing the following classes:

β-Lactams: amoxicillin, ampicillin, cloxacillin, dicloxacillin, oxacillin, penicillin, cefaclor, cefadroxil, cefalexin and cefazolin.

Macrolides: azithromycin, clarithromycin, erythromycin and tylosin.

Lincosamides: lincomycin.

Pleuromutilins: tiamulin.

Phenicols: chloramphenicol, florfenicol and thiamphenicol.

Quinolones and fluoroquinolones: ciprofloxacin, danofloxacin, difloxacin, enrofloxacin, marbofloxacin, norfloxacin, ofloxacin, sarafloxacin, flumequine and oxolinic acid.

Tetracyclines: chlortetracycline, doxycycline, oxytetracycline and tetracycline.

Sulfonamides: sulfachloropyridazine, sulfaclozine, sulfadiazine, sulfadimethoxine, sulfadimidine, sulfadoxine, sulfaguanidine, sulfamerazine, sulfamethizole, sulfamethoxazole, sulfamethoxypyridazine, sulfamonomethoxine, sulfamoxole, sulfapyridine, sulfaquinoxaline, sulfathiazole and sulfisoxazole.

Diaminopyrimidines: trimethoprim.

For solid samples, residues other than β-lactams were extracted following Gago-Ferrero et al. [Ref S3]. β-lactam extraction was based on Meklati et al. [Ref S4] with minor modifications. Three grams of solid sample were placed into a 50-mL polypropylene tube and 30 mL

phosphate-buffer solution at pH 8.5 were added. Samples were shaken for 15 min, followed by addition of 1 mL acetonitrile and vortex mixing. Samples were centrifuged at 4000 rpm for 10 min and the supernatant was transferred to a new tube. Samples were subsequently cleaned by solid-phase extraction using Strata-X cartridges (200 mg/6 mL). Cartridges were preconditioned with methanol and phosphate buffer, samples were loaded, cartridges washed and dried under vacuum, and analytes eluted using 5 mL acetonitrile. Samples were evaporated to dryness at 40°C and reconstituted before analysis in 300 µL of 0.1% formic acid:methanol containing 0.1% formic acid (95:5, v/v). Antibiotic residues in water were extracted following Galani et al. [Ref S5], using an initial water volume of 1 L and a final reconstitution volume of 0.25 mL. Residues were quantified using liquid chromatography coupled with tandem mass spectrometry (LC-MS/MS). Sulfonamides were quantified using sulfadiazine-d4 as internal standard. Other residues were quantified using matrix-matched spiked samples.

S4. Trace-element analysis

Total concentrations of copper (Cu), zinc (Zn), nickel (Ni), lead (Pb), chromium (Cr) and cadmium (Cd) were measured. Soil, manure and vegetable samples were oven-dried at 40°C. A 100–150 mg portion of powdered sample was calcined at 450°C for 4 h to eliminate organic matter, followed by complete digestion with HF, HNO_3 and $HClO_4$ according to ISO 14869-1. Water samples were analysed after filtration without prior mineralization. Cu and Zn concentrations were determined using a PerkinElmer NexION 300X ICP-MS with ^103Rh as internal standard. Quality control was ensured using certified reference materials EnviroMAT EP-L-3, ES-H-2 and SRLS-5. DTPA-extractable Cu and Zn were additionally assessed in soil and manure samples following Lindsay and Norvell [Ref S6]. Samples were extracted using a solution containing 5 mM DTPA, 10 mM $CaCl_2$ and 100 mM triethanolamine buffered at pH 7.3, using soil-to-solution and manure-to-solution ratios of 1:2 and 1:25, respectively. Mixtures were stirred for 2 h and centrifuged at 1000 × g. Supernatants were filtered through 8-µm ashless medium-speed filter paper and analysed using flame atomic absorption spectrophotometry.

S5. Statistical analyses

Data analysis and visualization were performed in R. A Ct threshold of 27 was used as the limit for target detection. Relative abundance was computed in proportion to the 16S rRNA gene using the comparative Ct method. The number of detected ARGs and their relative abundance were summarized by sample and matrix. At the sample level, ARG relative abundance was summarized as the arithmetic mean of detected ARG abundances, excluding missing values. At the matrix level, mobile genetic elements (MGEs) were excluded from ARG abundance calculations and the mean was calculated from these sample-level averages, with 95% confidence intervals estimated by nonparametric bootstrap resampling of composite samples. Confidence intervals were not reported for matrices represented by a single sample (n = 1).ARG composition was compared using Bray–Curtis dissimilarities and visualized using non-metric multidimensional scaling.

Fold changes were calculated for paired samples to explore three contrasts:

1. fresh versus aged compost;
2. soil two versus four months after amendment;
3. amended versus paired unamended soils.

Because comparisons of composting and time since amendment each involved a single sample pair, these analyses were considered descriptive and hypothesis-generating. Linear regressions were used to explore relationships between trace-element concentrations and either the number or relative abundance of ARGs. Given the limited and unbalanced sampling design, inferential analyses were interpreted cautiously.

B- Supplementary Tables

Table S1. Description of environmental samples

Sample	Site	Matrix	Description
S1	A	Manure	Pig slurry
S2	A	Manure	Poultry litter
S3	A	Soil	Pig-slurry-amended soil
S4	A	Soil	Poultry-litter-amended soil
S5	A	Soil	Unamended agricultural control; synthetic fertilizer
S6	B	Water	Composite stream-water sample
S7	C	Manure	Fresh mixed manure
S8	C	Manure	Six-month composted manure
S9	C	Soil	Unamended agricultural soil
S10	C	Soil	Four months after compost amendment
S11	C	Soil	Two months after compost amendment
S12	C	Vegetables	Pooled lettuce, coriander and cabbage
S13	D	Soil	Natural soil
S14	E	Soil	Natural soil
S15	F	Soil	Natural soil
S16	G	Soil	Natural soil

Table S2. ARGs detected uniquely in specific environmental matrices

List of ARGs detected exclusively in individual matrix categories among manure, amended soils, control soils, vegetables and water.

Assay	Gene	Family	Unique to		Assay	Gene	Family	Unique to
AY164	vanRA_1	VAN	Manure		AY90	ermA/ermTR	MLSB	Manure
AY17	aphA1/7	AMI	Manure		AY131	pbp5	BL	Manure
AY2	aacC2	AMI	Manure		AY132	pbp	BL	Manure
AY206	cmr	MDR	Manure		AY165	vanSA	VAN	Manure
AY212	mdtG_1	MDR	Manure		AY176	vanRC4	VAN	Manure
AY22	str	AMI	Manure		AY258	tetK	TET	Manure
AY250	tet32	TET	Manure		AY66	msrA_1	MLSB	Manure
AY259	tetQ	TET	Manure		AY380	vanSC_2	VAN	Control soils
AY260	tetH	TET	Manure		AY385	aac(6')-Iy	AMI	Control soils
AY267	tetX	TET	Manure		AY574	tetM	TET	Control soils
AY268	tetC_2	TET	Manure		AY437	blaPER	BL	Control soils
AY294	intI2_2	INT	Manure		AY613	blaOXA24	BL	Amended soils
AY299	tnpA_1	MGE	Manure		AY338	bla1	BL	Amended soils
AY325	tetR_1	TET	Manure		AY557	catA2	PHE	Amended soils
AY406	aac6-aph2	AMI	Manure		AY109	blaPSE	BL	Amended soils
AY41	cmlA_4	PHE	Manure		AY113	bla-L1	BL	Amended soils
AY426	apmA	AMI	Manure		AY115	cepA	BL	Amended soils
AY46	ermF_1	MLSB	Manure		AY177	vanRD	VAN	Amended soils
AY460	qnrS_1	QUI	Manure		AY188	nimE	Other	Amended soils
AY553	msrE	MLSB	Manure		AY421	aph3-viia	AMI	Amended soils
AY559	catB2	PHE	Manure		AY430	ampC_cefa	BL	Amended soils
AY56	ermB_1	MLSB	Manure		AY431	blaSME	BL	Amended soils
AY568	tet39	TET	Manure		AY448	blaHERA	BL	Amended soils
AY75	lnuA_1	MLSB	Manure		AY451	blaLEN	BL	Amended soils
AY98	ampC_1	BL	Manure		AY469	fosX	Other	Amended soils
AY110	blaOXA10_1	BL	Manure		AY541	vga(A)LC_1	MLSB	Amended soils
AY30	catB8	PHE	Manure		AY570	tet38	TET	Amended soils
AY399	aac(6)-im	AMI	Manure		AY95	qnrA	QUI	Amended soils
AY4	aacA/aphD	AMI	Manure		AY484	bexA/norM	MDR	Vegetable plants

Table S3. ARGs co-occurring in source and receiving matrices while absent from paired control soils

List of ARGs detected jointly in manure and associated receiving matrices while absent from the corresponding unamended control soil.

id_assay	Gene	Class	High-risk for human-health	Present in	Absent in
AY181	vanTG	VAN	no	S1&S3	S5
AY21	aadE	AMI	yes	S1&S3	S5
AY23	strA	AMI	no	S1&S3	S5
AY236	qacEΔ1_3	Other	no	S1&S3	S5
AY249	tet36_1	TET	no	S1&S3	S5
AY264	tetO_2	TET	yes	S1&S3	S5
AY269	tetS	TET	no	S1&S3	S5
AY274	tetPB_1	TET	no	S1&S3	S5
AY281	tetM_2	TET	no	S1&S3	S5
AY284	dfrA1_1	TRI	no	S1&S3	S5
AY331	aadA2_3	AMI	no	S1&S3	S5
AY368	tetM_3	TET	no	S1&S3	S5
AY423	aph4-ia	AMI	no	S1&S3	S5
AY487	cefa_qacelta	MDR	no	S1&S3	S5
AY523	Tn3	MGE	no	S1&S3	S5
AY533	ermB_2	MLSB	no	S1&S3	S5
AY534	ermD	MLSB	no	S1&S3	S5
AY535	ermF	MLSB	no	S1&S3	S5
AY536	lnuB	MLSB	yes	S1&S3	S5
AY549	lnuF	MLSB	no	S1&S3	S5
AY551	mefB	MLSB	no	S1&S3	S5
AY579	dfrA1	TRI	yes	S1&S3	S5
AY580	dfrA15	TRI	yes	S1&S3	S5
AY594	dfrK	TRI	no	S1&S3	S5
AY65	mefA_1	MLSB	no	S1&S3	S5
AY549	lnuF	MLSB	no	S2&S4	S5
AY142	ttgB	Other	no	S8&S10	S9
AY162	vanHD	VAN	no	S8&S10	S9
AY204	sat4	Other	no	S8&S10	S9
AY236	qacEΔ1_3	Other	no	S8&S10	S9
AY265	tetM_1	TET	no	S8&S10	S9
AY274	tetPB_1	TET	no	S8&S10	S9
AY276	tetT	TET	no	S8&S10	S9
AY331	aadA2_3	AMI	no	S8&S10	S9
AY487	cefa_qacelta	MDR	no	S8&S10	S9
AY523	Tn3	MGE	no	S8&S10	S9
AY533	ermB_2	MLSB	no	S8&S10	S9
AY536	lnuB	MLSB	yes	S8&S10	S9
AY551	mefB	MLSB	no	S8&S10	S9
AY57	ermT_1	MLSB	yes	S8&S10	S9
AY581	dfra17	TRI	yes	S8&S10	S9
AY594	dfrK	TRI	no	S8&S10	S9
AY65	mefA_1	MLSB	no	S8&S10	S9
AY83	ermY	MLSB	no	S8&S10	S9
AY142	ttgB	Other	no	S8&S11	S9
AY162	vanHD	VAN	no	S8&S11	S9
AY204	sat4	Other	no	S8&S11	S9
AY218	qacEΔ1_1	Other	no	S8&S11	S9
AY236	qacEΔ1_3	Other	no	S8&S11	S9
AY265	tetM_1	TET	no	S8&S11	S9
AY274	tetPB_1	TET	no	S8&S11	S9
AY276	tetT	TET	no	S8&S11	S9
AY331	aadA2_3	AMI	no	S8&S11	S9
AY423	aph4-ia	AMI	no	S8&S11	S9
AY487	cefa_qacelta	MDR	no	S8&S11	S9
AY523	Tn3	MGE	no	S8&S11	S9
AY533	ermB_2	MLSB	no	S8&S11	S9
AY534	ermD	MLSB	no	S8&S11	S9
AY536	lnuB	MLSB	yes	S8&S11	S9
AY551	mefB	MLSB	no	S8&S11	S9
AY57	ermT_1	MLSB	yes	S8&S11	S9
AY593	dfrG	TRI	no	S8&S11	S9
AY594	dfrK	TRI	no	S8&S11	S9
AY7	aphA3_1	AMI	no	S8&S11	S9
AY83	ermY	MLSB	no	S8&S11	S9
AY142	ttgB	Other	no	S8&S12	S9
AY453	blaBEL	BL	no	S8&S12	S9
AY537	lnuC	MLSB	no	S8&S12	S9
AY551	mefB	MLSB	no	S8&S12	S9

Table S4. Antibiotic residues detected in environmental samples

Antibiotic traces detected. Values shown in grey designate concentrations expressed in µg/kg, whereas white cells represent measurements below the indicated detection limit. Only samples and antibiotics with at least one detectable value are included.

Antibiotic	Family	Samples				
		S1	S2	S8	S9	S11
Dicloxacillin	BL	<1,96	**15,4**	<1,96	<1,96	<1,96
Tiamulin	MLSB	**9,45**	<3,3	**26,4**	<3,3	<3,3
Oxolinic acid	QUI	**609**	<5,8	<5,8	<5,8	<5,8
Sulfamerazine	SUL	<2,8	<2,8	<2,8	**47,8**	**80,1**
Sulfamethizole	SUL	<3,0	<3,0	<3,0	<3,0	**35,7**

Table S5. Complete high-throughput qPCR dataset

Due to its large size, the complete dataset is provided separately as **Excel File S1**.

C- Supplementary Figures

Figure S1. Taxonomic-marker profiles across environmental samples

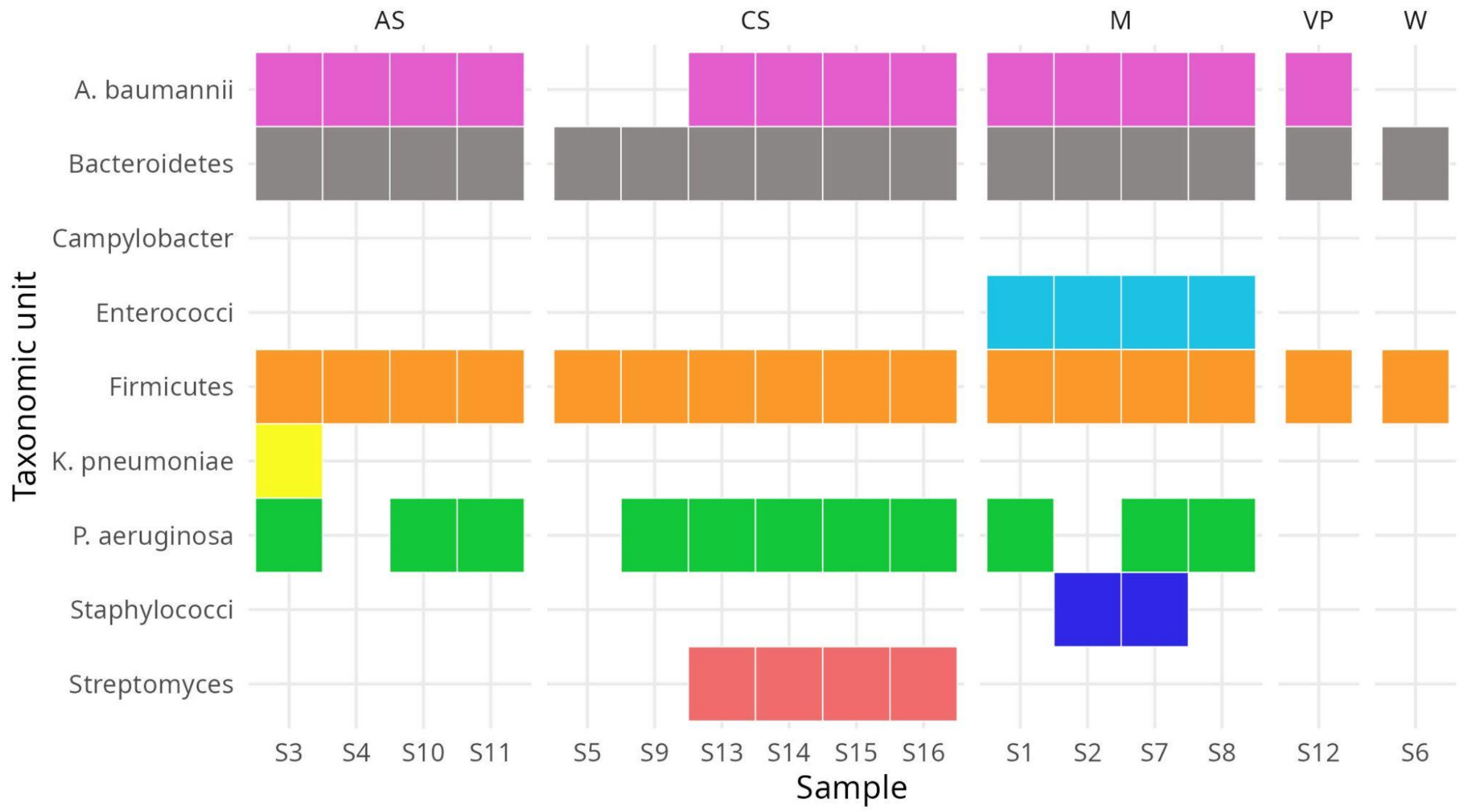

Occurrence of the targeted microbial taxonomic markers across individual samples. Among the nine targeted taxonomic groups, Campylobacter was not detected, whereas Bacteroidetes- and Firmicutes-associated markers were detected across samples. Streptomyces markers were detected in natural soils, and Enterococci-associated markers were associated mainly with manure samples. Because the taxonomic panel represents only a small targeted subset of microbial diversity, these results are descriptive and are not used to infer complete microbial-community composition. AS: Amended soils, CS: Control soils, M: Manure, VP: Vegetable plants, W: Water.

Figure S2. ARG distribution detected across different matrices

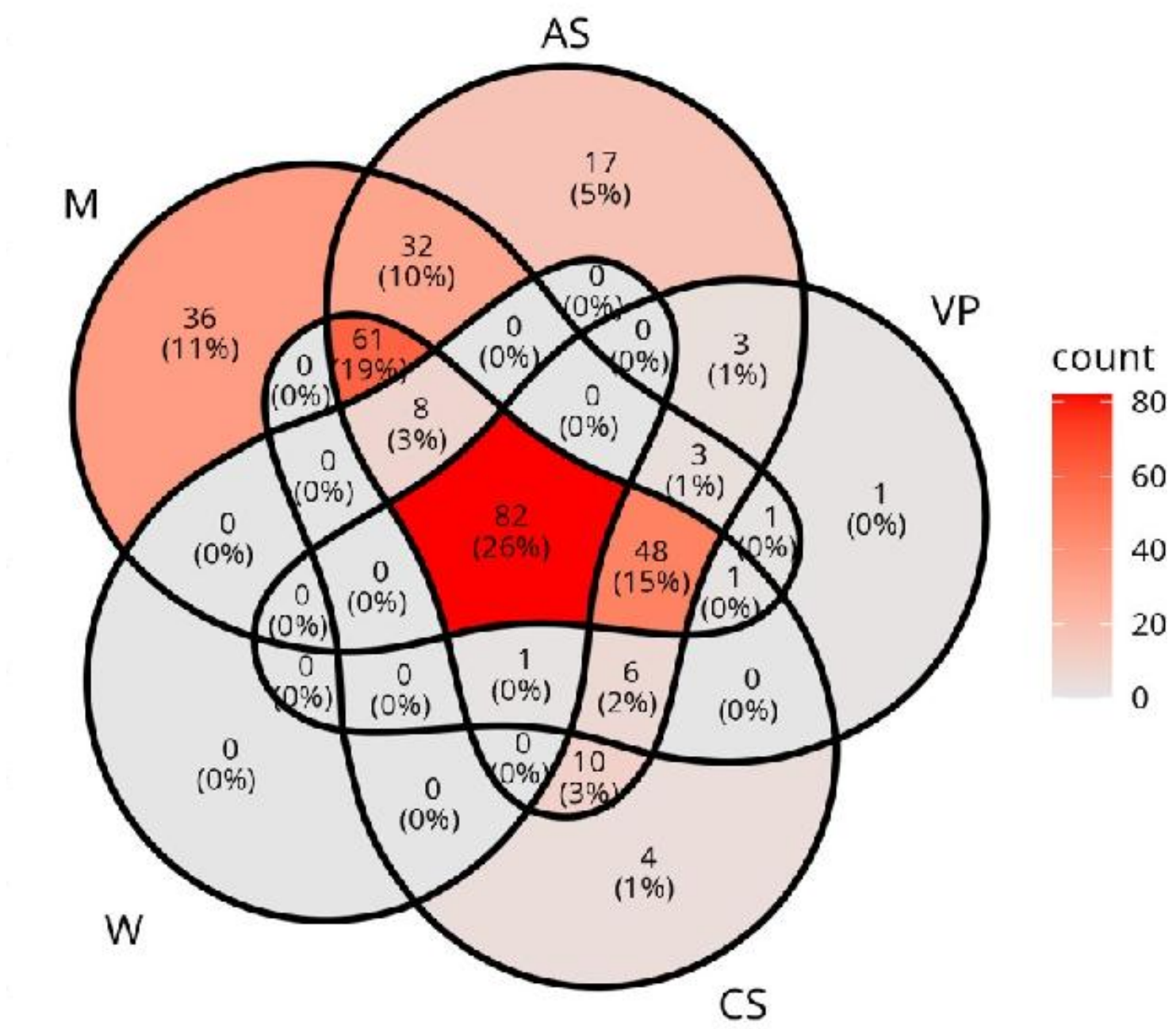

Venn diagram showing the distribution of antibiotic resistance genes (ARGs) detected across the different matrices, with overlaps indicating genes shared between matrices. AS: Amended soils, CS: Control soils, M: Manure, VP: Vegetable plants, W: Water.

Figure S3. ARG profiles in anthropized and natural control soils

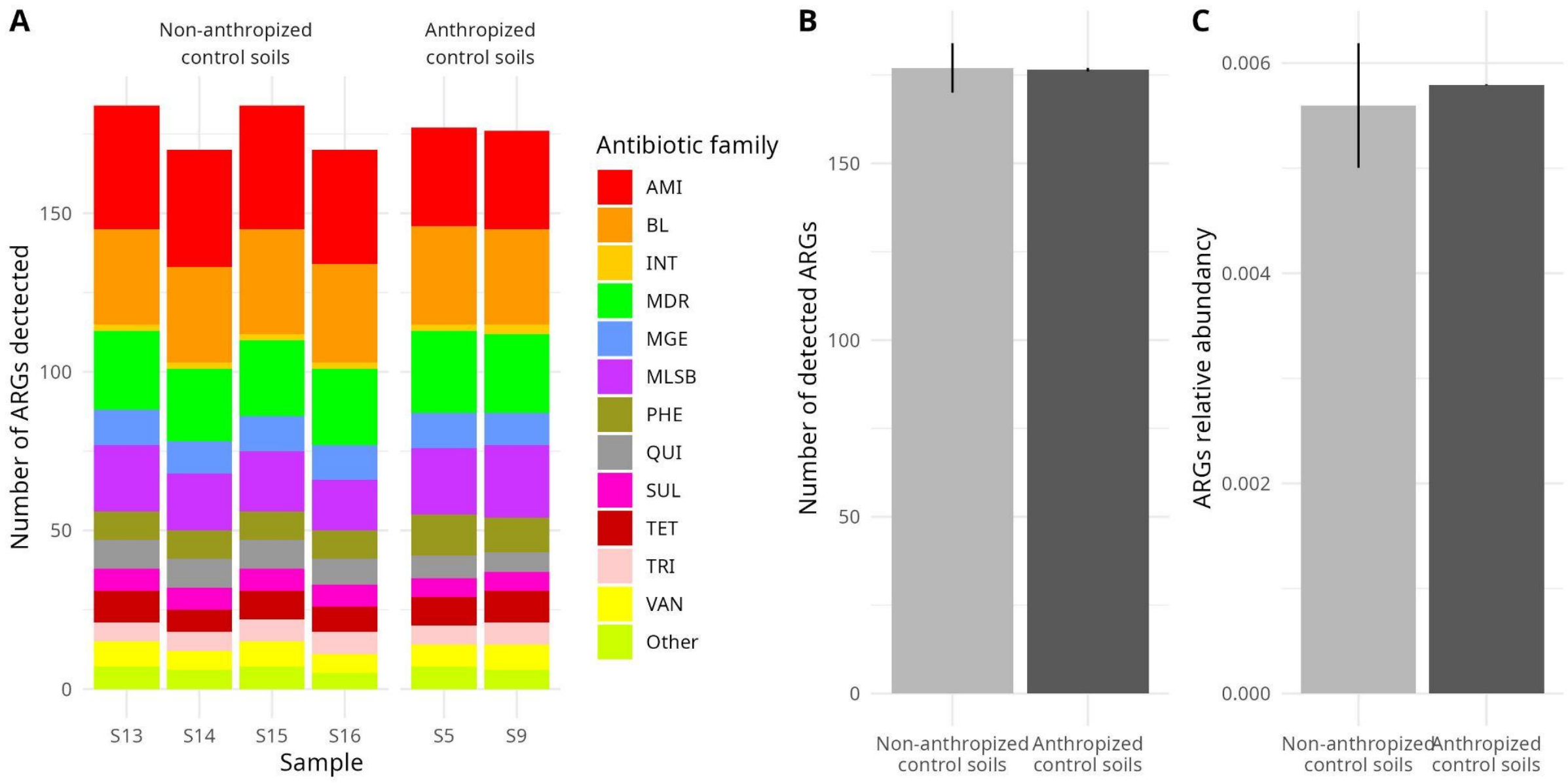

Comparison of (A) the number of detected ARGs by antibiotic resistance category in individual samples, (B) the mean number of detected ARGs, and (C) the mean relative abundance of ARGs between non-anthropized control soils (n = 4) and unamended agricultural control soils (n = 2). No clear difference was observed between the two control categories in this exploratory dataset.

Figure S4. Complete resistome profiles across environmental samples

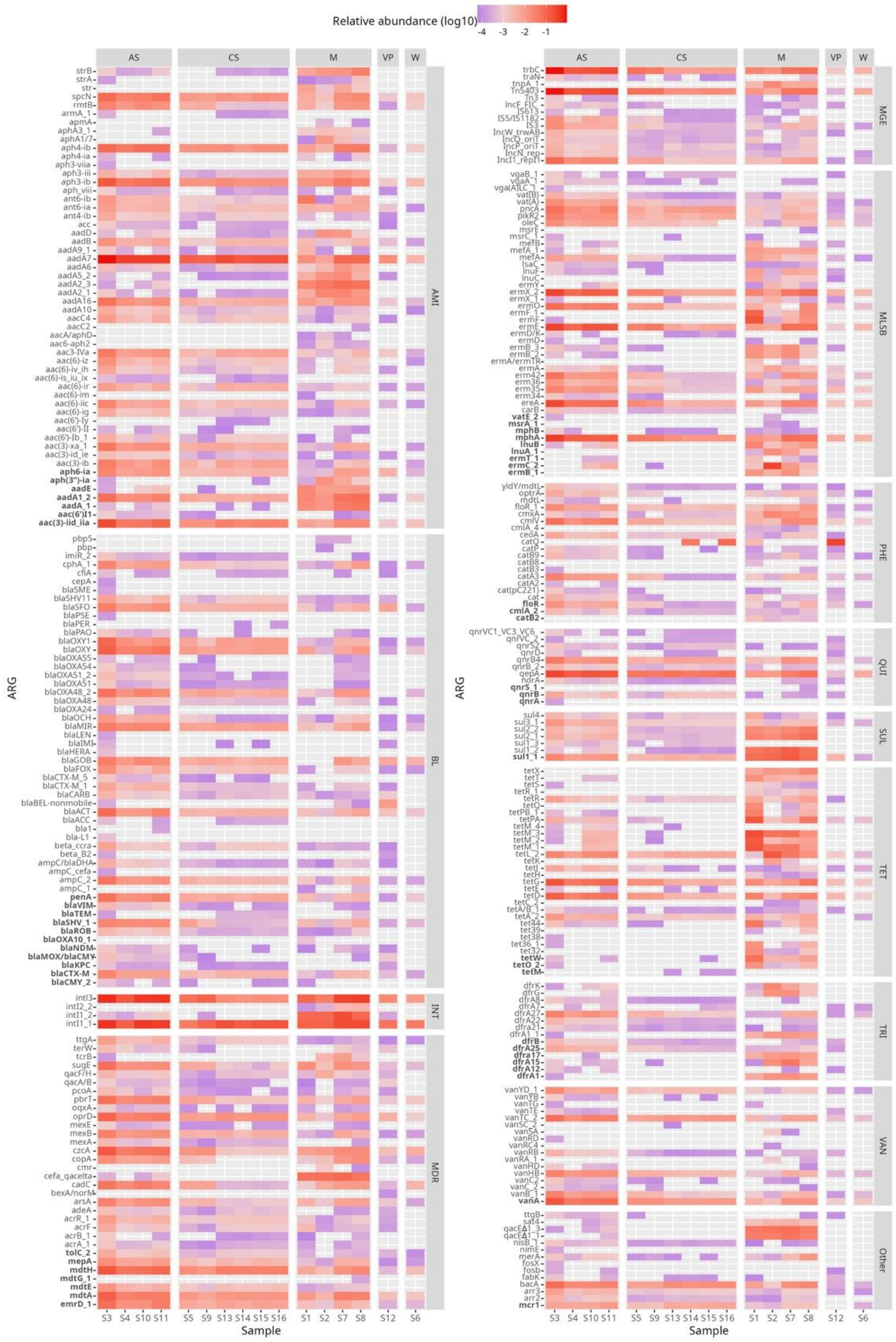

Heatmap showing the relative abundance of all 296 ARGs and 14 MGEs detected in at least one sample. Targets are grouped according to antibiotic-resistance class or MGE category. ARGs classified as high risk to human health according to Zhang et al. [12] are indicated. The main-text Fig. 3B is restricted to the 60 high-risk ARGs to improve readability and emphasize One Health relevance.

Figure S5. Exploratory fold changes in ARG abundance between paired samples

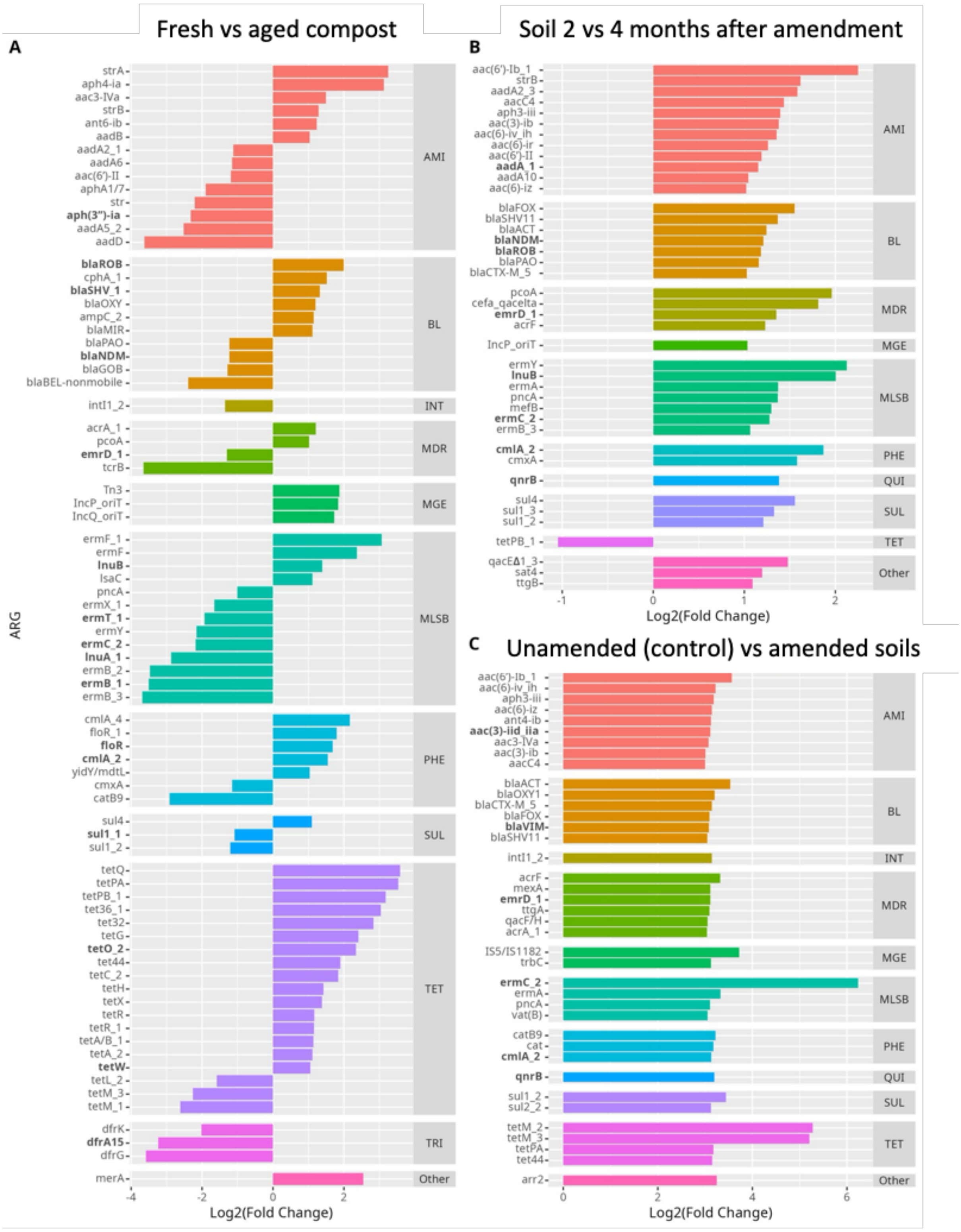

Fold changes in ARG relative abundance between (A) fresh and six-month composted manure, (B) soils sampled two and four months after amendment, and (C) paired unamended and amended soils. Fold changes are log_2-transformed. Only targets showing at least twofold differences are displayed. ARGs classified as high risk to human health are highlighted.ARG relative abundance was approximately 5-fold higher on average in amended than paired

unamended soils. Comparisons A and B each involve a single pair and should therefore be considered descriptive, hypothesis-generating observations rather than estimates of general composting or temporal effects.

Figure S6. Trace-element concentrations across environmental samples

	Nitot	Cutot	Pbtot	Zntot	Crtot	Cdtot	ZnDTPA	CuDTPA
**S1**	14,6	273,8	2	1716,7	9,1	1,01	1176,8	150,3
**S2**	7,4	112,7	0,2	647	7,2	0,36	273,2	35,2
**S3**	38,4	94,7	14,5	220,2	56,1	0,3	5,1	2,8
**S4**	32,8	83,4	10,1	190,3	47	0,22	1,8	1,7
**S5**	36,8	87,7	13,3	216,1	60	0,11	5,0	3,0
**S6**	0,4	2,1	0,5	6,4	0,8	0,03	NA	NA
**S7**	15,8	127,4	0,2	788,2	8,5	0,44	421,7	34,3
**S8**	19,9	125,6	0,7	715,5	17,8	0,41	414,2	27,2
**S9**	179	63,8	3	130,9	211,9	0,12	2,9	1,7
**S10**	163,7	65,3	2,4	131	234,7	0,12	6,9	1,3
**S11**	155,5	63,6	2	134,7	281,6	0,1	8,4	2,1
**S12**	NA	5	NA	50	NA	NA	NA	NA
**S13**	245,6	71,8	4,4	108,3	395,5	0,38	0,8	0,9
**S14**	189,3	37,4	5,9	97	236,2	0,01	0,6	1,5
**S15**	355,3	105,5	6,8	113,7	585,3	0,09	0,8	2,3
**S16**	48,1	28,4	10,1	199,3	67,4	0,15	1,6	1,1

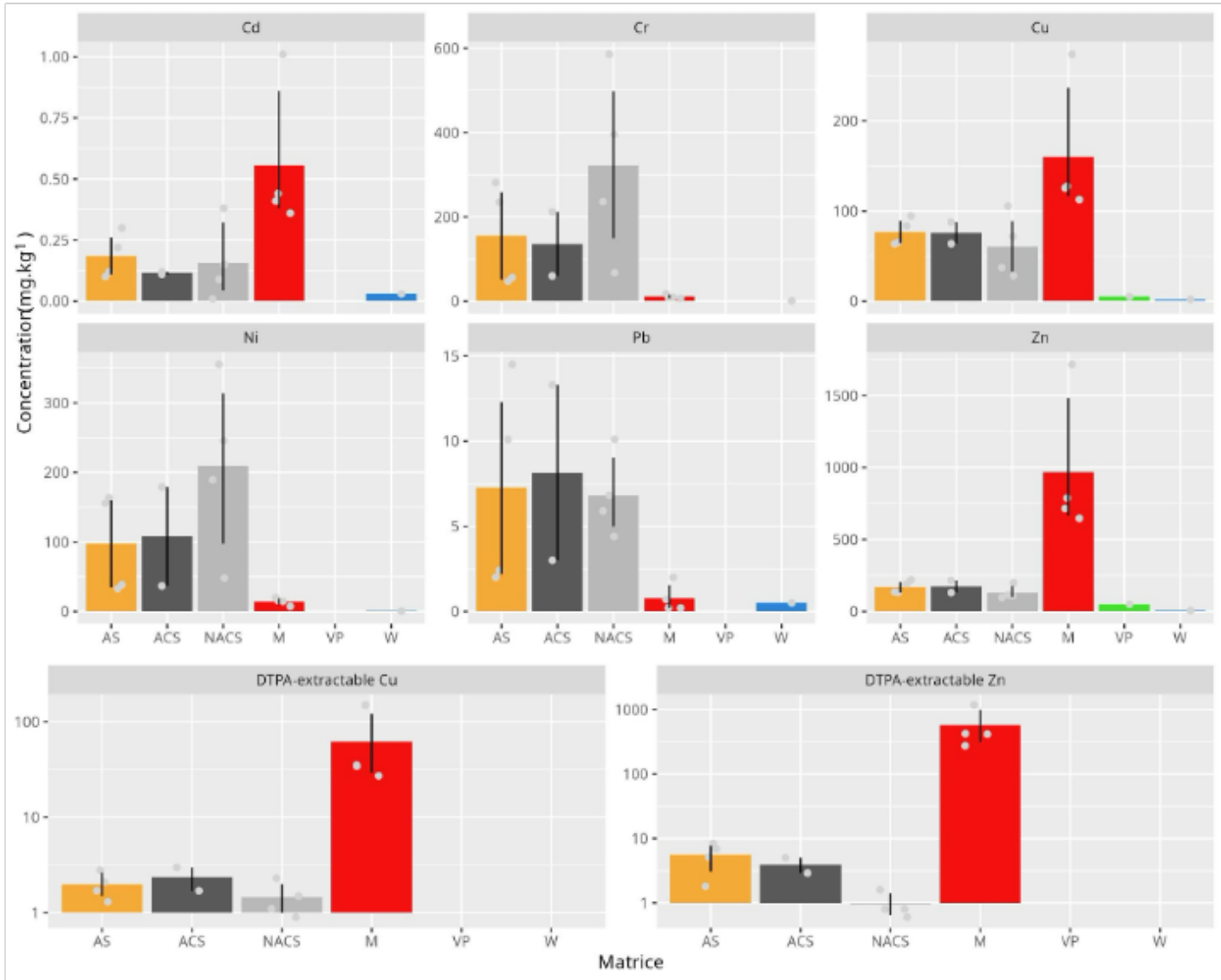

Total concentrations of Cd, Cr, Cu, Ni, Pb and Zn and DTPA-extractable Cu and Zn across individual samples and matrices. Bars represent matrix means ± SD and points represent individual measurements. AS, amended soils; ACS, anthropized control soils; NACS, natural control soils; M, manure; VP, vegetable plants; W, water.

Figure S7. Occurrence of antibiotic residues and abundance of corresponding ARGs

Dicloxacillin

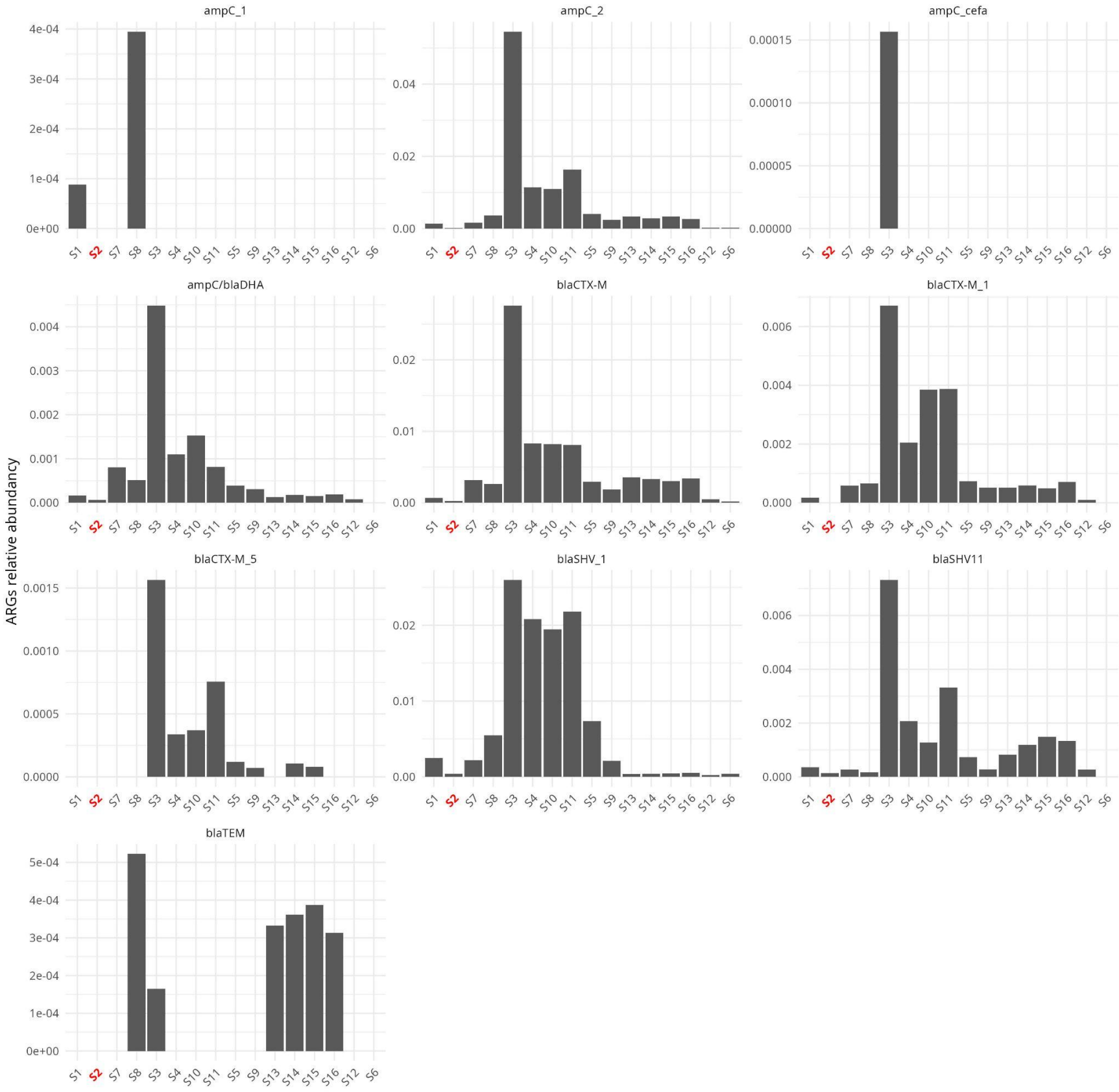

Oxolinic acid

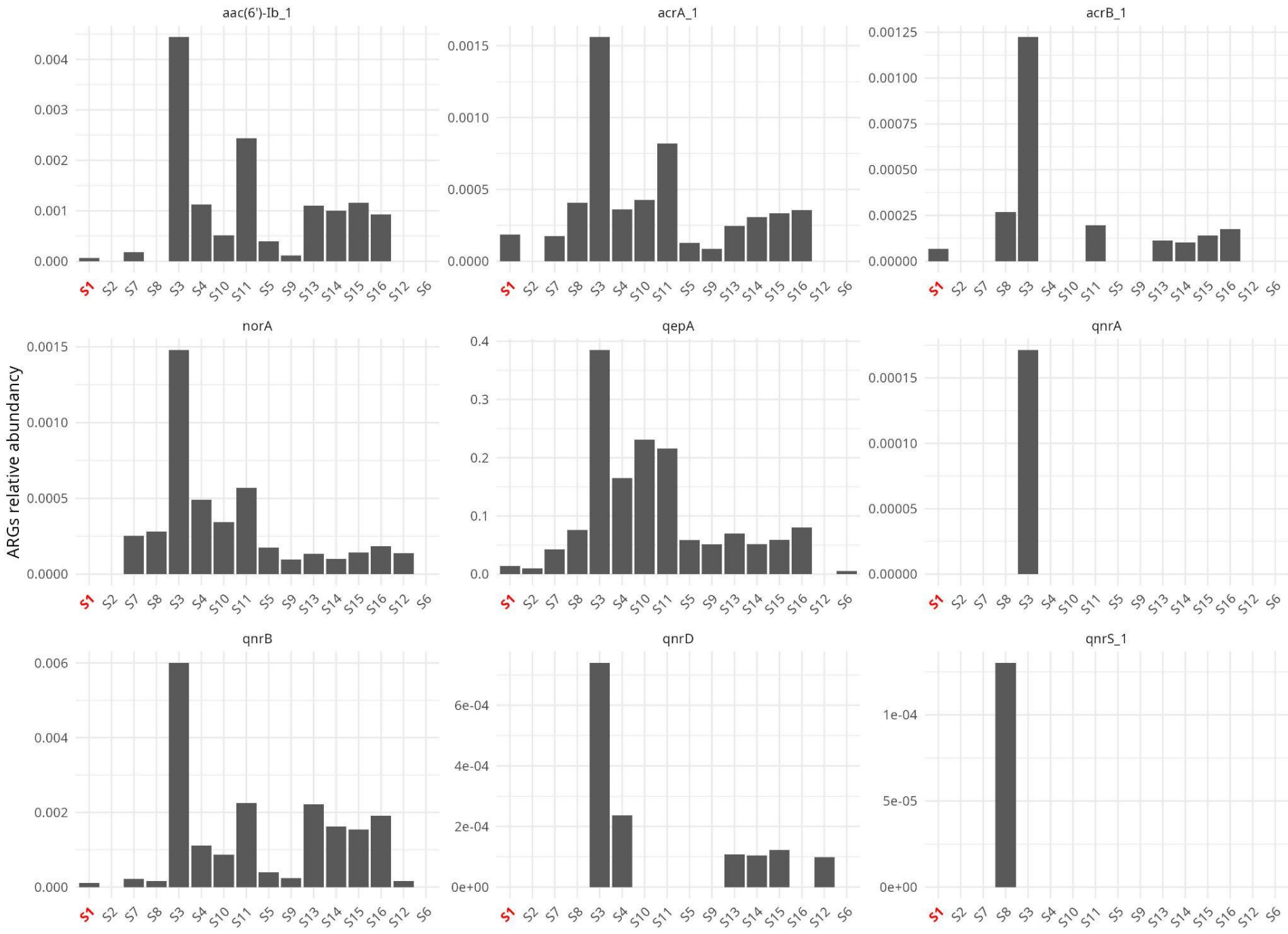

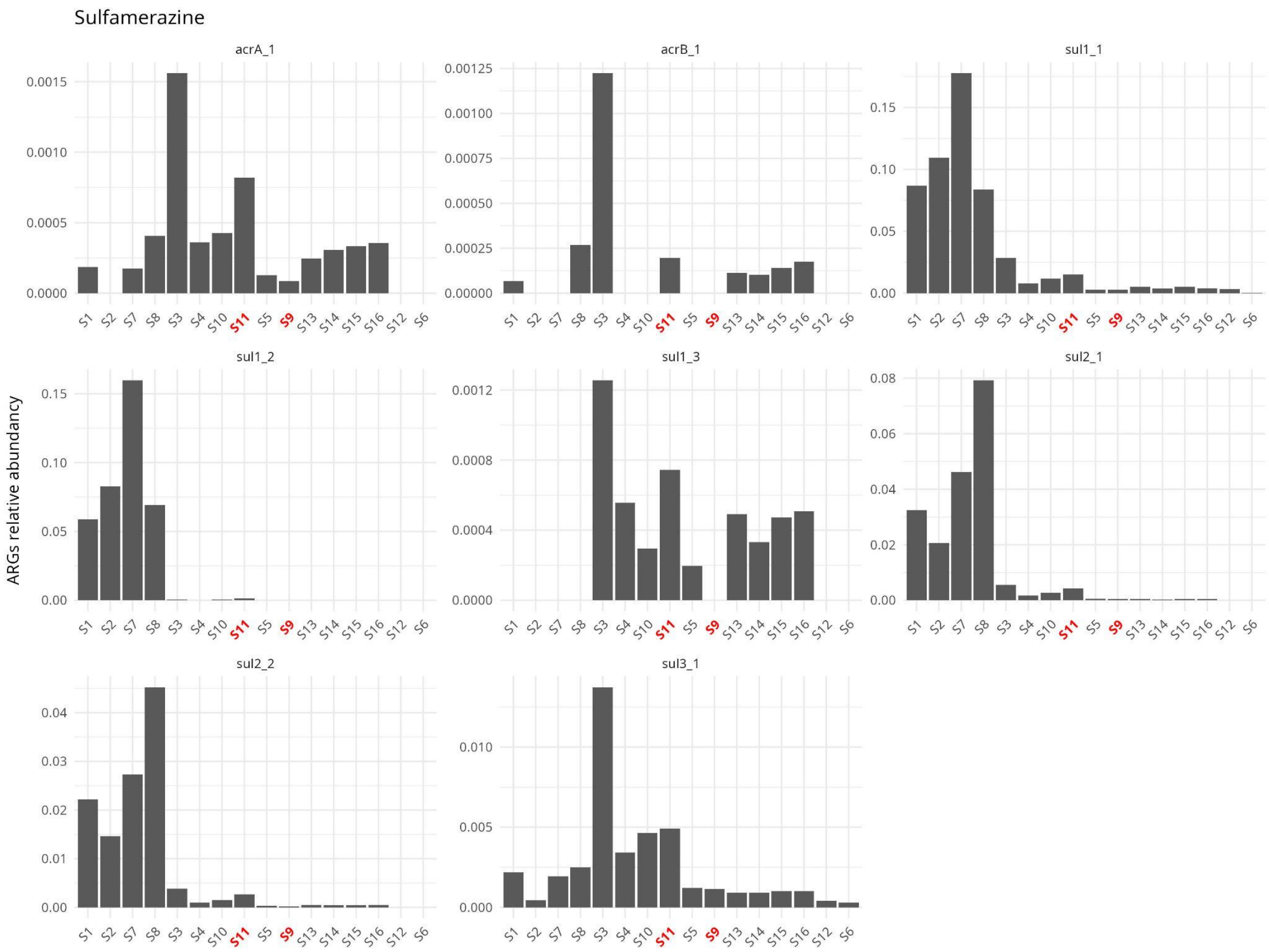

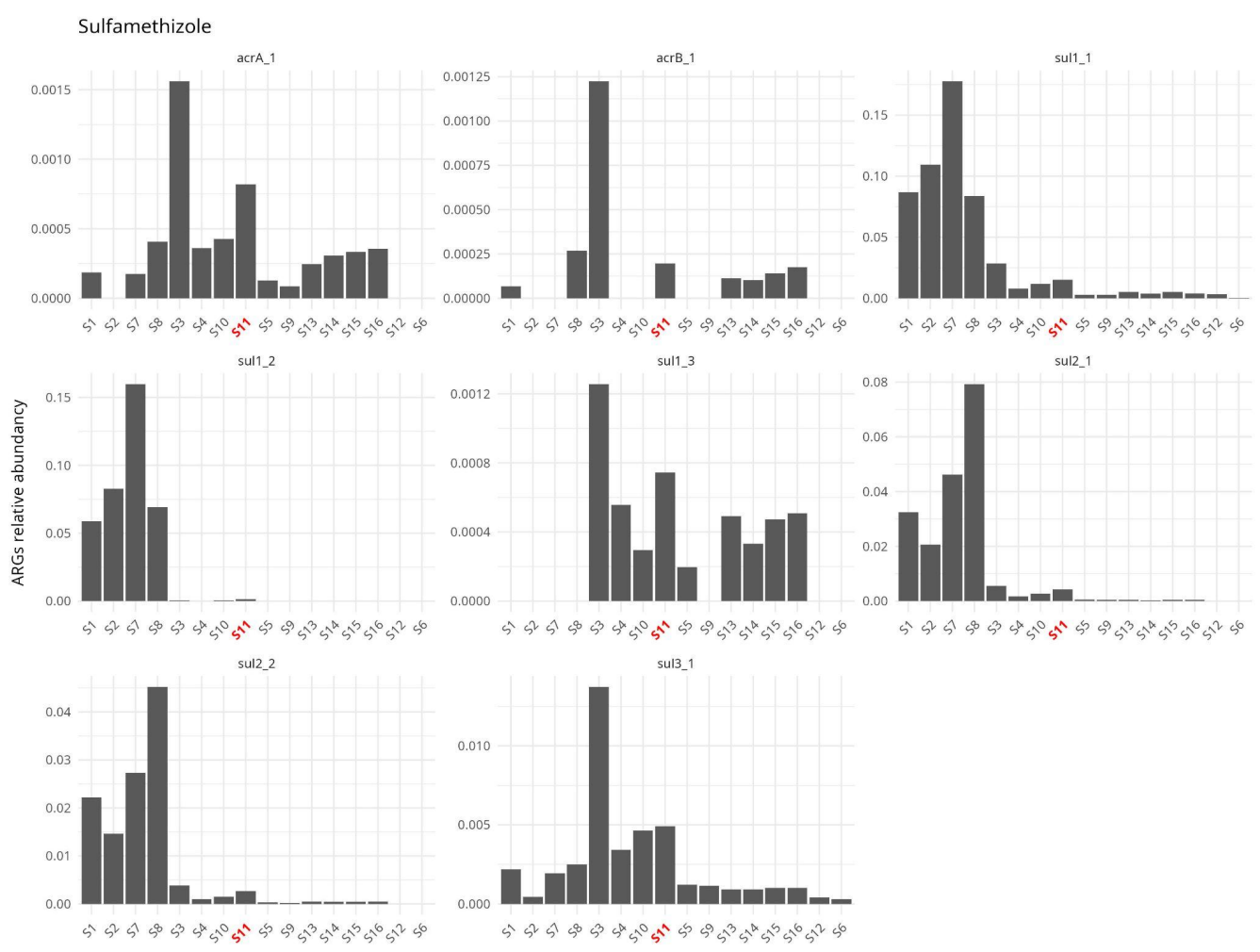

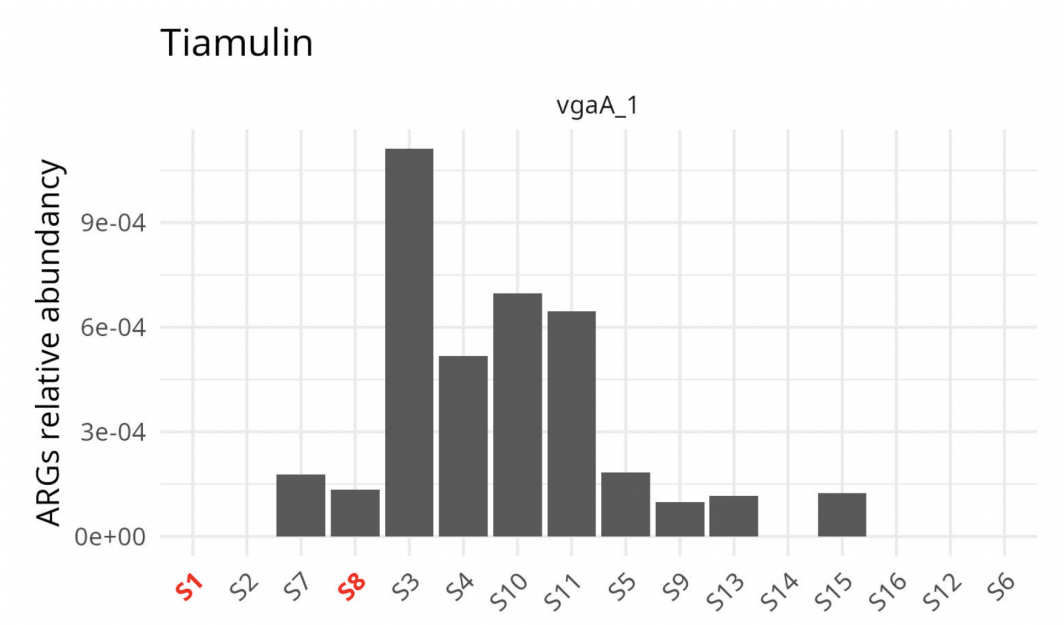

Relative abundance of ARGs associated with the antibiotic compounds detected in the investigated samples. Samples containing detectable residues are indicated in red. Because antibiotic residues were detected in only five samples, this analysis is descriptive and no causal relationship between residue occurrence and ARG abundance is inferred.

Figure S8. Exploratory relationships between Cu/Zn concentrations and ARG richness and relative abundance

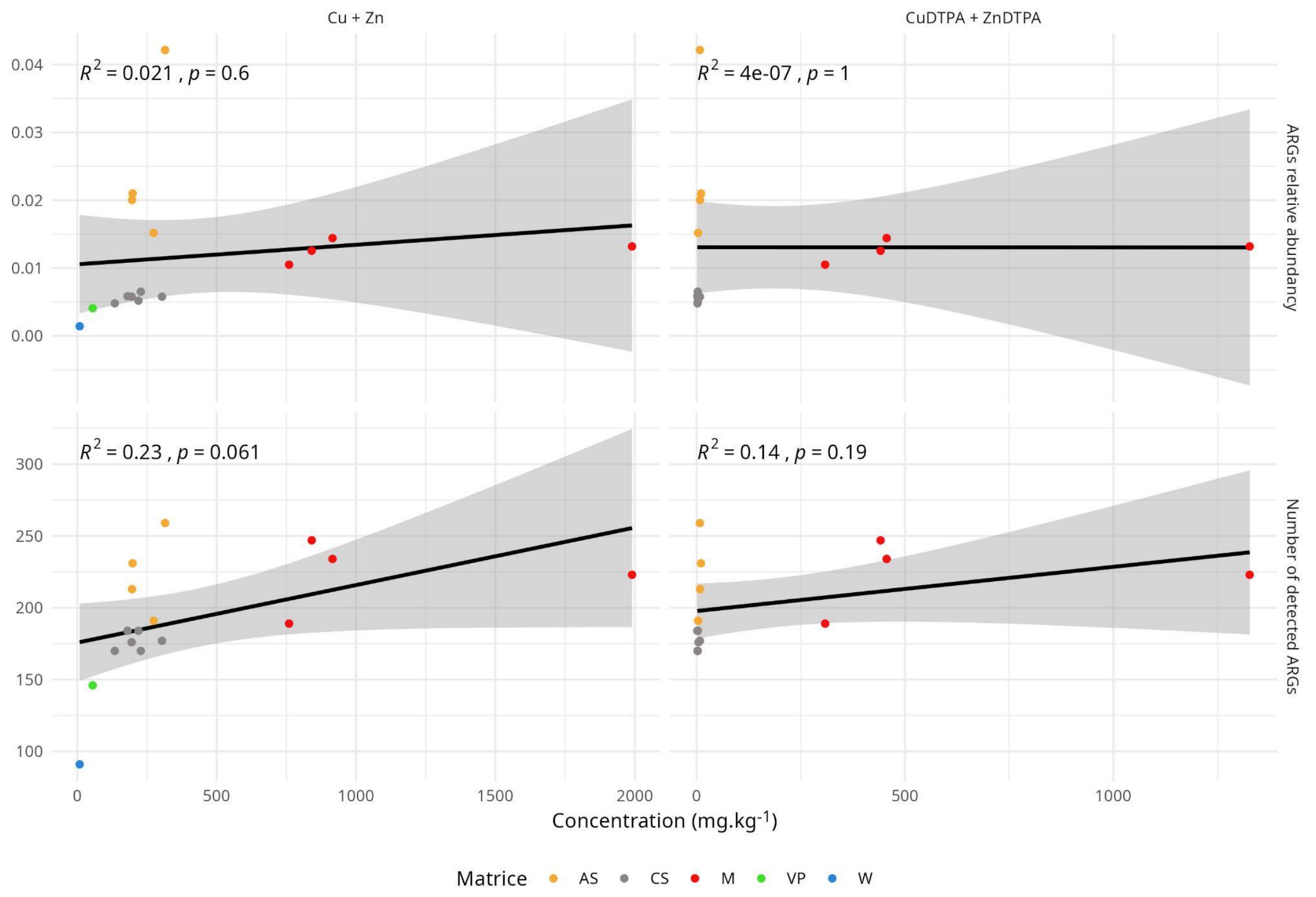

Linear relationships between total Cu + Zn or DTPA-extractable Cu + Zn concentrations and (A) ARG relative abundance and (B) number of detected ARGs. Shaded areas represent 95% confidence intervals around fitted regression lines. No statistically significant associations were detected. Given the small sample size, absence of a significant relationship should not be interpreted as evidence that Cu or Zn does not contribute to ARG selection or persistence.

D- Supplementary References

- Ref S1: Stedtfeld RD, Guo X, Stedtfeld TM, Sheng H, Williams MR, Hauschild K, Gunturu S, Tift L, Wang F, Howe A, Chai B, Yin D, Cole JR, Tiedje JM, Hashsham SA. Primer set 2.0 for

highly parallel qPCR array targeting antibiotic resistance genes and mobile genetic elements. FEMS Microbiol Ecol. 2018 Sep 1;94(9):fiy130. doi: 10.1093/femsec/fiy130. PMID: 30052926; PMCID: PMC7250373.

- Ref S2: T.D. Schmittgen, K.J. Livak, Analyzing real-time PCR data by the comparative C(T) method, Nat. Protoc. 3 (2008) 1101–1108.

- Ref S3. Gago-Ferrero P, Borova V, Dasenaki ME, Thomaidis NS. Simultaneous determination of 148 pharmaceuticals and illicit drugs in sewage sludge based on ultrasound-assisted extraction and liquid chromatography–tandem mass spectrometry. Anal Bioanal Chem. 2015;407:4287–4297.

- Ref S4. Meklati FR, Panara A, Hadef A, Meribai A, Ben-Mahdi MH, Dasenaki ME, Thomaidis NS. Comparative Assessment of Antibiotic Residues Using Liquid Chromatography Coupled with Tandem Mass Spectrometry (LC-MS/MS) and a Rapid Screening Test in Raw Milk Collected from the North-Central Algerian Dairies. Toxics. 2022 Jan 5;10(1):19. doi: 10.3390/toxics10010019. PMID: 35051061; PMCID: PMC8781432.

- Ref S5. Galani A, Alygizakis N, Aalizadeh R, Kastritis E, Dimopoulos MA, Thomaidis NS. Patterns of pharmaceuticals use during the first wave of COVID-19 pandemic in Athens, Greece as revealed by wastewater-based epidemiology. Sci Total Environ. 2021 Dec 1;798:149014. doi: 10.1016/j.scitotenv.2021.149014. Epub 2021 Jul 21. PMID: 34325143; PMCID: PMC8294694.

- Ref S6. Lindsay WL, Norvell WA. Development of a DTPA soil test for zinc, iron, manganese, and copper. Soil Sci Soc Am J. 1978;42:421–428.
